# Plasma homocysteine levels and risk of congestive heart failure or cardiomyopathy: A Mendelian randomization study

**DOI:** 10.3389/fcvm.2023.1030257

**Published:** 2023-01-26

**Authors:** Xinyi Wang, Zhuo Chen, Wende Tian, Jie Zhang, Qiuyi Li, Jianqing Ju, Hao Xu, Keji Chen

**Affiliations:** ^1^National Clinical Research Center for Chinese Medicine Cardiology, Xiyuan Hospital, China Academy of Chinese Medical Sciences, Beijing, China; ^2^Graduate School, Beijing University of Chinese Medicine, Beijing, China

**Keywords:** two-sample Mendelian randomization, genome-wide association study, plasma homocysteine levels, congestive heart failure, cardiomyopathy

## Abstract

**Background:**

Although observational studies have demonstrated associations between elevated plasma homocysteine levels and the risk of cardiovascular diseases, controversy remains.

**Objective:**

This study investigated the causal association of plasma homocysteine levels with congestive heart failure and cardiomyopathy risk.

**Methods:**

We performed a two-sample Mendelian randomization (MR) study of congestive heart failure (*n* = 218,792), cardiomyopathy (*n* = 159,811), and non-ischemic cardiomyopathy (*n* = 187,152). Genetic summary data on the association of single-nucleotide polymorphisms with homocysteine were extracted from the most extensive genome-wide association study of 44,147 individuals. MR analyses, including the random-effect inverse variance-weighted (IVW) meta-analysis, weighted median, simple median, maximum likelihood, penalized weighted median, MR-PRESSO, and MR-Egger regression, were used to estimate the associations between the selected single-nucleotide polymorphisms and congestive heart failure or cardiomyopathy.

**Results:**

The MR analyses revealed no causal role of higher genetically predicted plasma homocysteine levels with congestive heart failure risk (random-effect IVW, odds ratio [OR] per standard deviation (SD) increase in homocysteine levels = 1.753, 95% confidence interval [CI] = 0.674–4.562, *P* = 0.250), cardiomyopathy (random-effect IVW, OR per SD increase in homocysteine levels = 0.805, 95% CI = 0.583 to 1.020, *P* = 0.189), or non-ischemic cardiomyopathy (random-effect IVW, OR per SD increase in homocysteine levels = 1.064, 95% CI = 0.927–1.222, *P* = 0.379). The results were consistent with other analytical methods and sensitivity analyses.

**Conclusion:**

Genetically predicted homocysteine level was not associated with congestive heart failure or cardiomyopathy risk. It is unlikely that homocysteine-lowering therapy decreases the incidence or improves the outcomes of congestive heart failure and cardiomyopathy.

## 1. Introduction

Congestive heart failure is a significant public health problem that causes considerable morbidity and mortality, accounting for over 1 million hospitalizations annually (1). Prevention of congestive heart failure by identifying risk factors or indicators is crucial. Risk factors for congestive heart failure include age, sex, coronary artery disease, myocardial infarction, hypertension, diabetes mellitus, and obesity (2–7). Perturbed myocardial energetics participate in mechanisms leading to heart failure as myocardial adenosine trisphosphate production is reduced by 30–40% (8). A recent study suggested that the cardiomyopathy burden across the world increased substantially from 1990 to 2019, and constituted a considerable global public health problem with increasing prevalence, deaths, and disability-adjusted life years over recent decades (9). The interrelationship between congestive heart failure and cardiomyopathy is complex yet close. Heart failure is highly influenced by heritability, and there are nearly 100 genes linked to inherited forms of cardiomyopathy; as clinically observed, heart failure is frequently accompanied by cardiomyopathy and they present with similar symptoms (10). Heart failure might be the common outcome of an individual’s heart disease; however, many cases of heart failure initially present with different forms of cardiomyopathy. Therefore, it is hypothesized that intervention at the cardiomyopathic stage may attenuate or prevent subsequent heart failure initiation and progression.

Homocysteine, a sulfhydryl-containing non-proteinogenic amino acid, is physiologically critical for cell cycle progression and maintenance of cellular homeostasis (11). Elevated plasma homocysteine concentrations are associated with increased risks of various cardiovascular diseases (CVD), including congestive heart failure (12, 13). Experimental studies underscored that the myocardium is uniquely susceptible to homocysteine injury (14). However, the causal relationship is uncertain, as some scholars questioned the conclusion that a high homocysteine level is not an independent risk factor for CVD due to the potential confounding factors in observational studies (15). It is unclear if high homocysteine levels are mechanistic risk factors for congestive heart failure or only risk indicators without any direct effects on the myocardium.

Mendelian randomization (MR) is an alternative approach to inferring the causality of lifelong risk factors (exposure) on diseases (outcome) using genetic variants as instrumental variables (IVs) (16). In this study, we performed MR analyses to assess the associations between genetically predicted plasma homocysteine levels with congestive heart failure and cardiomyopathy risk. We also explored the causal association between homocysteine and non-ischemic cardiomyopathies including hypertrophic cardiomyopathy, diabetic cardiomyopathy, arrhythmogenic cardiomyopathy, and cardiomyopathy associated with rare genetic diseases.

## 2. Materials and methods

This study was conducted according to STROBE-MR guidelines (17), as in Supplementary Table 1. Formal ethical approval is not required as this is an analysis of publicly available, deidentified and summarized data.

### 2.1. Study design

**FIGURE 1:**
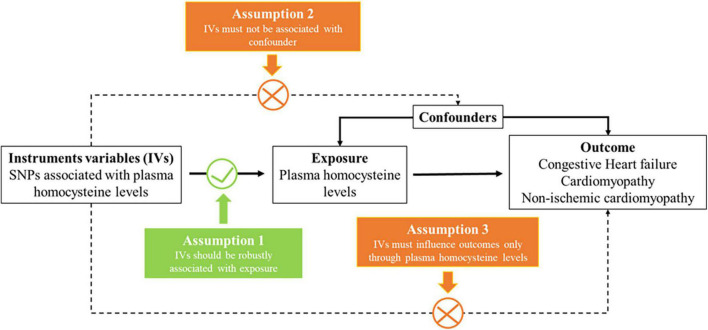
Three key assumptions underlying Mendelian randomization study design. SNP, single nucleotide polymorphism.

We designed a two-sample MR (TSMR) analysis to evaluate the causal effect of plasma homocysteine on diseases. The MR approach seeks IVs based on three principal assumptions (Figure 1; 18). First, IVs are strongly associated with the risk factor of the exposure (homocysteine). Second, IVs should not be associated with confounders. Finally, IVs only affect the outcomes (congestive heart failure, cardiomyopathy or non-ischemic cardiomyopathy) through the homocysteine pathway and not other pathways.

### 2.2. Data sources

**FIGURE 2:**
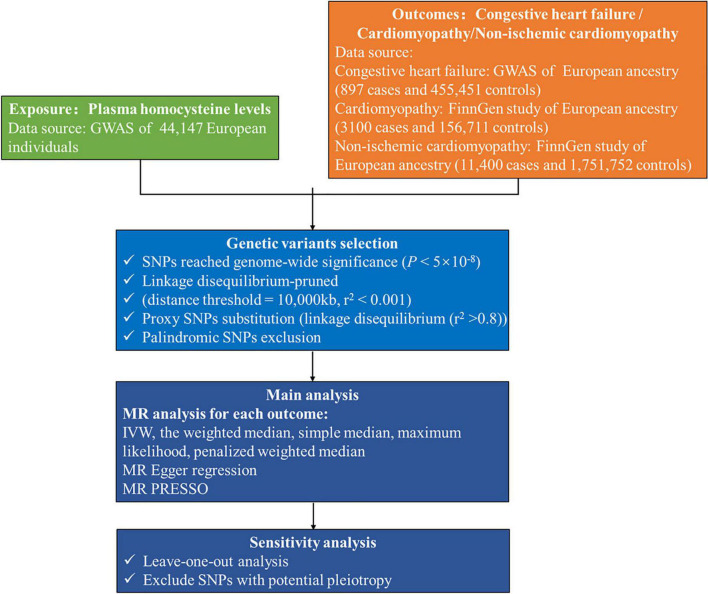
Flow chart of this Mendelian randomization study. GWAS, genome-wide association study; IVW, inverse-variance weighted; MR, Mendelian randomization; MR PRESSO, MR pleiotropy residual sum and outlier; SNP, single-nucleotide polymorphism.

We accessed the publicly available genome-wide association study (GWAS) summary statistics provided by the NHGRI-EBI GWAS Catalog^1^ or the Integrative Epidemiology Unit GWAS database.^2^ The exposure in this study was genetically predicted plasma homocysteine levels. We selected genetic variants associated with plasma homocysteine levels in a meta-analysis that included data from ten independent cohorts of European ancestry, with up to 44,147 individuals (19). Genetic variants associated with congestive heart failure were obtained from the most extensive published GWAS which contained 897 congestive heart failure cases and 455,451 controls in the GWAS Catalog (20). We selected GWAS summary statistics from the FinnGen cohort on the Integrative Epidemiology Unit Open GWAS Project database for cardiomyopathy and non-ischemic cardiomyopathy. The FinnGen study includes an expanding repository of genomic and clinical data emanating from a nationwide network of Finnish biobanks.^3^ For cardiomyopathy, we used the GWAS with the specific ID “finn-b-I9_CARDMYO” consisting of 3,100 cases, 156,711 controls and 16,380,196 genotyped single-nucleotide polymorphisms (SNPs). Publicly available data for non-ischemic cardiomyopathy were available from a GWAS with the specific ID “finn-b-I9_NONISCHCARDMYOP,” involving 11,400 cases and 175,752 controls. There was no overlap between the participants included in the GWAS for homocysteine and these outcomes. The datasets used in MR analysis include individuals of European ancestry to reduce selection bias and improve the robustness. The analytical procedure is shown in Figure 2.

### 2.3. Selection of genetic IVs

Single-nucleotide polymorphisms significantly associated with homocysteine levels were selected as IVs (*P* < 5.0 × 10^–8^). These SNPs were further linkage disequilibrium (LD)-pruned (distance threshold = 10,000 kb, *r*^2^ < 0.001) to ensure independence among the genetic variants (21). If the selected SNPs were not collected in the GWAS of congestive heart failure or cardiomyopathy, proxy SNPs in the LD (*r*^2^ > 0.8) were chosen for substitution. Subsequently, we removed any palindromic SNPs with minor allele frequencies above 0.3 to ensure that the effects of the SNPs on the exposure corresponded to the same allele as did their effects on diseases (22). To minimize potential weak instrument bias, we considered an F-statistic of at least 10 as sufficient for performing an MR analysis. The F statistics can be calculated as *F* = $\frac{R^{2}\,\left(N-1-k\right)}{\left(1-R^{2}\right)\,k}$, where *N* stands for the sample size, *k* stands for the number of IVs, and *R*^2^ stands for the percentage of the variation explained by the SNPs (23). *R*^2^ was derived from the original study or calculated according to the derived summary statistics in line with what has been described previously, which can be calculated as *R*^2^ = $2\times \left(1-m\,i\,n\,o\,r\,a\,l\,l\,e\,l\,e\,f\,r\,e\,q\,u\,e\,n\,c\,y\right)\times m\,i\,n\,o\,r\,a\,l\,l\,e\,l\,e\,f\,r\,e\,q\,u\,e\,n\,c\,y\times \left(\frac{\beta }{S\,E\times \sqrt{N}}\right)$^2^ (24).

### 2.4. Statistical analysis

We harmonized the summary exposure and outcome data based on a previously described method (25). The random-effect inverse variance weighted (IVW) was employed as the primary MR analysis to evaluate the causal effect between plasma homocysteine levels and congestive heart failure, cardiomyopathy, or non-ischemic cardiomyopathy, which assumes the absence of invalid genetic instruments (26). The weighted median, simple median, maximum likelihood and penalized weighted median methods were also employed. Compared with the IVW, these methods are more robust for individual genes with strongly outlying causal estimates and generate a consistent causal effect estimate when valid IVs exceed 50% (27). The MR-Egger method was used to detect directional pleiotropy, violating the above assumptions (28). Directional pleiotropy was assessed by evaluating the deviation of MR-Egger intercepts; a value that differs from zero indicates that the IVW estimate is biased (29). Cochran’s *Q* test was applied to assess the heterogeneity of estimates of individual genetic variability. We also inspected potential directional pleiotropy based on the asymmetry of the funnel plots. MR-PRESSO (MR Pleiotropy RESidual Sum and Outlier) was applied to validate the results in the IVW model, which detected and corrected for horizontal pleiotropy by removing outliers (30). The association was deemed causal when at least three methods provided consistent results, which reduces the risk of false-positive interpretations (31).

For sensitivity analysis, a leave-one-out sensitivity analysis was conducted by removing a single variant from the analysis each time to determine whether a single SNP disproportionately affected the association. We excluded the SNPs significantly associated with potential confounders by searching genome-wide traits (*P* < 5 × 10^–8^) using the Phenoscanner website^4^ (32).

The odds ratios (ORs) and corresponding 95% confidence intervals (CIs) of outcomes were scaled to a one-standard-deviation (SD) increase in genetically predicted homocysteine. A two-sided *P* < 0.05 was considered statistically significant for MR analyses. All analyses were performed using the “TwoSampleMR” and “MR-PRESSO” packages in the R software environment.

## 3. Results

### 3.1. IVs selection and validation

We obtained three IVs for congestive heart failure, 12 for cardiomyopathy, and 12 for non-ischemic cardiomyopathy. The details of the characteristics of all independent IVs in this study are displayed in Supplementary Table 2. F statistics for all SNPs used in this study were all above 10, indicating that they were robust instruments. We used the intercept term to estimate the exposures from MR-Egger regression and found that no horizontal pleiotropic pathway existed in our TSMR analysis (congestive heart failure: MR-Egger intercept = −0.1369, *P* = 0.257; cardiomyopathy: MR-Egger intercept = −0.0278, *P* = 0.290; non-ischemic cardiomyopathy: MR-Egger intercept = 0.0047, *P* = 0.687).

### 3.2. Results from TSMR

**FIGURE 3:**
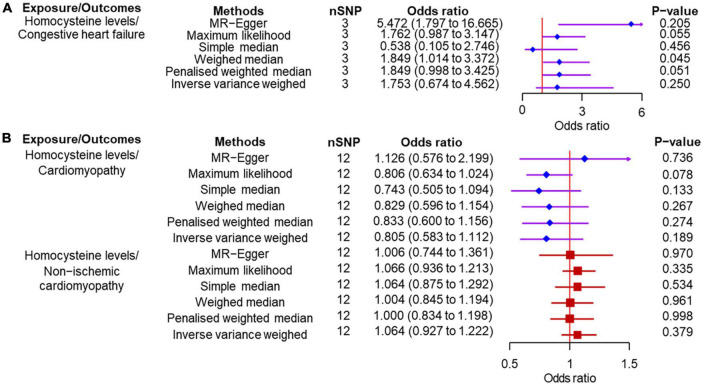
Two-sample Mendelian randomization of plasma homocysteine levels and the risk of diseases. **(A)** Congestive heart failure. **(B)** Cardiomyopathy and non-ischemic cardiomyopathy. CI, confidence interval; SNP, single nucleotide polymorphism.

**FIGURE 4:**
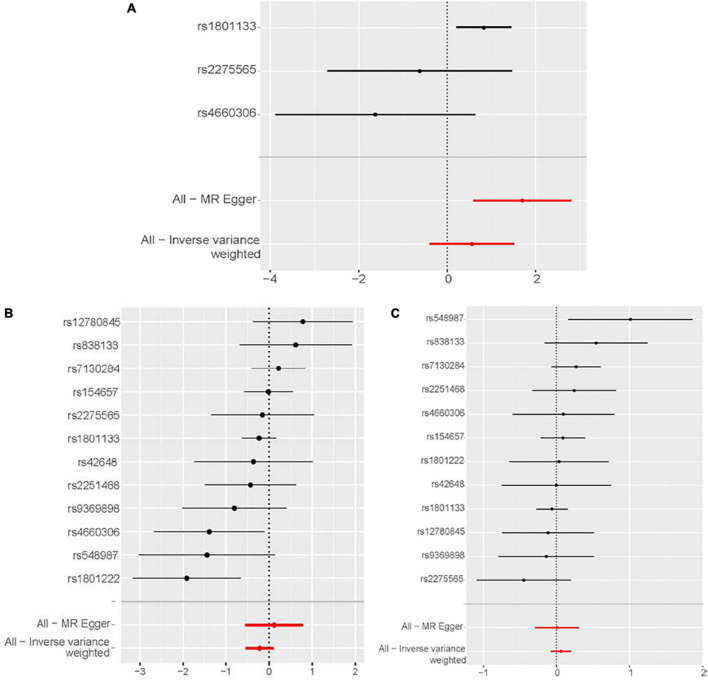
Forest plot of the potential effects of plasma homocysteine level–associated SNPs on outcomes. **(A)** Congestive heart failure. **(B)** Cardiomyopathy. **(C)** Non-ischemic cardiomyopathy. CI, confidence interval; IVW, inverse variance-weighted; MR, Mendelian randomization.

According to the IVW analysis results, for each SD increase in genetically predicted homocysteine levels, the OR was 1.753 (95% confidence interval (CI), 0.674–4.561; *P* = 0.250) for congestive heart failure, 0.805 (95% CI, 0.583–1.020; *P* = 0.189) for cardiomyopathy, and 1.064 (95% CI, 0.927–1.222; *P* = 0.379) for non-ischemic cardiomyopathy (Figure 3). The weighted median, simple median, maximum likelihood, and penalized weighted median methods yielded almost consistent results, except the MR-Egger regression results. Based on prespecified causality adjudication rules (31). These results suggest that genetically predicted plasma homocysteine levels are not associated with congestive heart failure, cardiomyopathy, or non-ischemic cardiomyopathy. The causal effect estimates of genetically determined homocysteine on the risk of congestive heart failure and cardiomyopathy are shown in Figure 4. No significant heterogeneity was detected using Cochran’s Q statistics among SNPs of homocysteine (congestive heart failure: *Q* = 5.532, *P* = 0.063; non-ischemic cardiomyopathy: *Q* = 12.751, *P* = 0.310, respectively), except for cardiomyopathy (*Q* = 20.478, *P* = 0.039). The funnel plots revealed an absence of directional pleiotropy, with a symmetrical distribution of variants effects (Supplementary Figure 1). No outlier was observed in the MR-PRESSO test regarding homocysteine-cardiomyopathy and homocysteine-non-ischemic cardiomyopathy MR analysis, and the SNPs of homocysteine-congestive heart failure are insufficient for MR-PRESSO test (only three SNPs).

### 3.3. Results from sensitivity analysis

**FIGURE 5:**
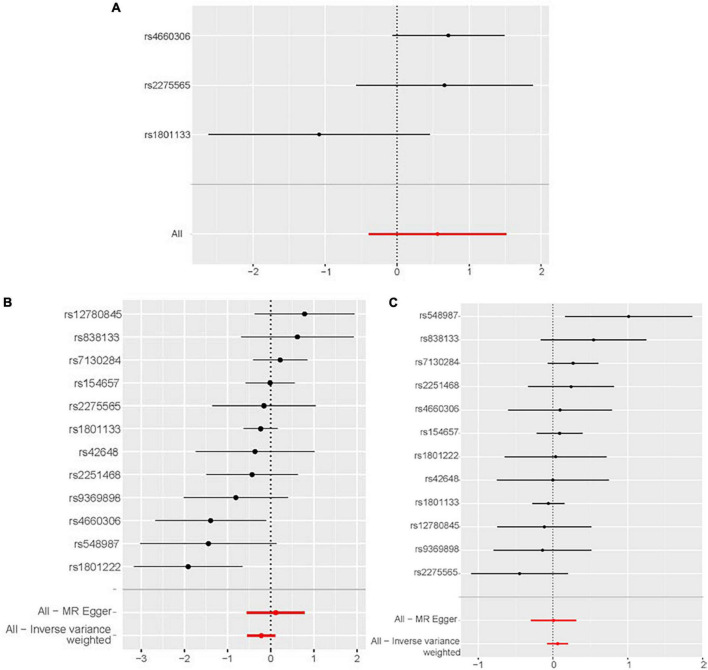
Sensitivity analyses using the leave-one-out approach for the association of plasma homocysteine level with outcomes. **(A)** Congestive heart failure. **(B)** Cardiomyopathy. **(C)** Non-ischemic cardiomyopathy. CI, confidence interval; IVW, inverse variance-weighted; MR, Mendelian randomization.

The leave-one-out analysis showed that the negative results for the relationship between homocysteine and congestive heart failure, cardiomyopathy and non-ischemic cardiomyopathy were not driven by any individual SNP exclusion, confirming the lack of associations (Figure 5).

The Phenoscanner database revealed one SNP associated with confounding factors for congestive heart failure and five for cardiomyopathy and non-ischemic cardiomyopathy, including BMI (33), cholesterol (34), hemoglobin concentration (35), coronary heart disease, and blood pressure (36; Supplementary Table 3). After excluding these pleiotropic SNPs, similar results were observed (Supplementary Table 4), and no significant directional pleiotropy (Supplementary Table 5) was found. No significant heterogeneity was detected, except for SNPs on cardiomyopathy (*Q* = 15.842, *P* = 0.014).

## 4. Discussion

We evaluated the relationship between plasma homocysteine level and congestive heart failure or cardiomyopathy risk using TSMR. We found that genetically predicted homocysteine level is unlikely to be a causal determinant of congestive heart failure, cardiomyopathy, or non-ischemic cardiomyopathy risk.

Homocysteine is a common amino acid that directly damages the vascular endothelium (37). Several lines of evidence suggest the associations between elevated plasma homocysteine levels and increased congestive heart failure risk. Prospective data from the community-based prospective Framingham Study analyzing 2491 adults demonstrated an increased incidence of congestive heart failure in individuals with elevated homocysteine levels (13). A recent observational study found that the homocysteine level was positively correlated with left ventricular end-diastolic diameter and New York Heart Association grade, and was negatively correlated with left ventricular ejection fraction (38). Increased homocysteine levels are prevalent in heart failure with reduced ejection fraction and heart failure with preserved ejection fraction (39). Clinical studies suggested that hyperhomocysteinemia (i.e., fasting plasma homocysteine > 10 μmol/l) is related to the incidence and severity of chronic heart failure (40, 41). A recent meta-analysis drew similar conclusions (42). Cardiomyopathy is the most frequent genetic cause of congestive heart failure owing to untreated and uncontrolled systolic dysfunction at a later stage. Although the number of studies exploring the association between homocysteine and cardiomyopathy is limited, there were sufficient hypotheses. The experimental evidence supports the findings that myocardium is uniquely susceptible to homocysteine-induced injury and high homocysteine levels increase the risk of cardiomyopathy (43, 44). In addition, selective homocysteine-lowering gene transfer potently attenuates pressure overload-induced cardiomyopathy, improves infarct healing, attenuates remodeling, and enhances diastolic function after myocardial infarction in mice (45, 46).

Although much evidence shows strong associations, randomized controlled trials (RCTs, the most powerful method of demonstrating the etiology hypothesis) showed contradictory results. Folate and vitamin B12 are commonly used to reduce homocysteine, although this effect is not selectively targeted. Several RCTs attempting to reduce serum homocysteine concentrations with folate supplementation (thereby reducing the detrimental effect of hyperhomocysteinemia on CVD) have not met their expectations (47). The third update of the Cochrane review reported uncertain effects of homocysteine-lowering interventions in preventing cardiovascular events, suggesting that hyperhomocysteinemia should not be regarded as an independent risk factor (48). RCTs of homocysteine-lowering as an intervention to treat cardiomyopathy are lacking; however, folate treatment in diabetic mice with hyperhomocysteinemia did not alleviate the development of diabetic cardiomyopathy (49). Though homocysteine levels can be used as biomarkers to improve the predictive ability of CVD prediction models, the diagnostic accuracy of the working characteristic curve only slightly improved when homocysteine was added to the traditional risk factors used for prediction in several cohort studies (50). The initial and extended follow-up of the B-PROOF trial indicated that folic acid and vitamin B12 supplementation did not affect CVD (51). The renal Hope-2 study showed that active treatment with B6, B12, and folic acid lowered homocysteine levels in participants with chronic kidney disease but did not reduce cardiovascular risk, and more participants in the active treatment group were hospitalized for heart failure (52). Based on current evidence, elevated homocysteine levels are unlikely to serve as independent risk factors for heart failure or even CVD.

Studies suggested that elevated plasma homocysteine levels were associated with an increased risk of other CVD, including coronary heart disease (53), hypertension (54), atrial fibrillation (55), and stroke (56). Nevertheless, like our study, several MR studies failed to identify a causal relationship between elevated plasma homocysteine and CVD. Sun et al. performed an MR study suggesting plasma homocysteine levels were not causally associated with atrial fibrillation (24). Miao et al. conducted a TSMR study estimating the role of increased plasma homocysteine levels on the etiology of coronary heart disease and acute myocardial infarction and found no causal relationship (57). Borges et al. performed an MR analysis to assess the causal influence of homocysteine on systolic and diastolic blood pressure, and the findings did not corroborate the hypothesis that homocysteine has a causal role in blood pressure (58). Another MR analysis indicated that circulating total homocysteine was associated with small vessel stroke but not with other subtypes such as large artery stroke and cardioembolic stroke (59). Regarding the association between homocysteine and heart failure, the results are compelling. Consistent with our findings, an MR analysis that evaluated the association between genetically predicted homocysteine, vitamin B12 levels, and indistinguishable types of heart failure also showed negative results (60). Strauss et al. used MR to examine the associations of the methylene tetrahydrofolate gene and paraoxonase 1 gene variants as a proxy for lifelong exposure to high homocysteine and homocysteine-thiolactone concentrations with the development of heart failure in men aged ≤ 60 years, providing evidence that hyperhomocysteinemia is a causal factor for non-ischemic heart failure in dilated cardiomyopathy (61). Facing the results of lacking causality, there are several possible explanations. The association of homocysteine and congestive heart failure or cardiomyopathy, even CVD, shown in observed studies may result from confounding factors. Although these studies adjusted for some confounders, it is impossible to control unmeasured risk factors completely. In MR analysis, unmeasured confounding factors were equally distributed between the exposure and control groups to avoid the influence of possible confounding factors. The sample size might not be large enough in traditional observational studies to detect the exact association. The side effects of elevated homocysteine levels on CVD may be due not to homocysteine itself but to the downstream products of homocysteine metabolism. Moreover, the elevated plasma homocysteine may be a consequence of CVD. Aksoy et al. proposed a hypothesis that a diminished clearance rate caused by impaired renal function was a prominent pathophysiological mechanism in the elevation of homocysteine concentration in heart failure (62). Finally, we should recognize that research on the role of hyperhomocysteinemia in the occurrence and progression of heart failure should be performed under specific etiologic scenarios.

This TSMR analysis provides genetic evidence that homocysteine level is not significantly associated with congestive heart failure or cardiomyopathy risk, which supplements the existing evidence that homocysteine has no causal relationship with CVD. Strengths of the present study include the TSMR study design and the large sample size. Another strength is that though folic acid and B-vitamins supplementation normalize homocysteine levels, it is unwise to use them to prevent or treat heart failure and cardiomyopathy in clinical practice because they may provide no benefit. Previous MR analysis only investigated the association between homocysteine and indistinguishable types of heart failure, which may cause significant heterogeneity. A recent GWAS and MR analysis exploring the pathogenesis of heart failure suggested further MR analysis of heart failure subtypes to reduce the effect of heterogeneity (63). Though ischemic and non-ischemic congestive heart failure shares a similar clinical presentation, the underlying pathophysiological mechanisms differ. In this study, we precisely selected the congestive heart failure population for analysis and shed light on the relevance of homocysteine and heart failure. The following potential limitations also require attention. First, we did not conduct MR analyses stratified by other heart failure subtypes (e.g., heart failure with reduced ejection fraction and heart failure with preserved ejection fraction), and cardiomyopathy subtypes (e.g., hypertrophic, dilated, and restrictive cardiomyopathy) due to limited available summary data; nevertheless, MR analysis for homocysteine and non-ischemic cardiomyopathy was conducted to exclude the influence of ischemic cardiomyopathy caused by coronary atherosclerosis. Second, it is impossible to be certain that the variants used in this study do not have pleiotropic effects, despite a lack of evidence in favor of strong pleiotropy. Third, we cannot perform the reverse analysis because the GWAS for homocysteine is not publicly available. Fourth, the published data we used are summary-level statistics; therefore, we cannot examine any potential non-linear relationships or stratification effects that differ by age, gender or other conditions. Fifth, our study relied on genetic data conducted in a population primarily of European descent for greater genetic homogeneity, limiting the applicability of results for other ethnic backgrounds. In addition, the limited number of SNPs (especially in the analysis for congestive heart failure) may cause some bias. Nevertheless, we selected the GWAS with the largest sample size for the analysis. Finally, MR analysis provides evidence supporting a causal effect but does not directly demonstrate causation (58). We only revealed the relationship between homocysteine and diseases from a genetic point of view without involving other environmental factors.

## 5. Conclusion

This TSMR analysis revealed that genetically predicted homocysteine level was not associated with congestive heart failure or cardiomyopathy risk. It was plausible that simply reducing plasma homocysteine levels could not decrease the incidence or improve the outcomes of congestive heart failure and cardiomyopathy in clinical practice. More work is warranted to confirm our results further.

## Data availability statement

The datasets presented in this study can be found in online repositories. The names of the repository/repositories and accession number(s) can be found below: The NHGRI-EBI GWAS catalog (https://www.ebi.ac.uk/gwas) or the Integrative Epidemiology Unit (IEU) GWAS database (https://gwas.mrcieu.ac.uk/).

## Ethics statement

Ethical review and approval was not required for the study on human participants in accordance with the local legislation and institutional requirements. Written informed consent for participation was not required for this study in accordance with the national legislation and the institutional requirements.

## Author contributions

XW and HX conceived and designed the study. XW, ZC, and WT conducted the research. JZ, QL, and JJ analyzed the data. XW wrote the manuscript. ZC, HX, and KC reviewed and edited the manuscript. All authors read and approved the final manuscript.

## Footnotes

1
https://www.ebi.ac.uk/gwas

2
https://gwas.mrcieu.ac.uk/

3
https://finngen.gitbook.io/documentation/

4
http://www.phenoscanner.medschl.cam.ac.uk
